# Evaluation of the influenza-like illness surveillance system in Tunisia, 2012–2015

**DOI:** 10.1186/s12889-019-7035-3

**Published:** 2019-06-06

**Authors:** Rihab Yazidi, Wafa Aissi, Hind Bouguerra, Mariem Nouira, Ghassen Kharroubi, Latifa Maazaoui, Mokhtar Zorraga, Naima Abdeddaiem, Sadok Chlif, Awatef El Moussi, Mohamed Ali Ben Hadj Kacem, Mohamed Ali Snoussi, Wissem Ghawar, Makram Koubaa, Lauren Polansky, Margaret McCarron, Mohamed Boussarsar, Khaled Menif, Slim Amine, Jalila Ben Khelil, Mounir Ben Jemaa, Jihene Bettaieb, Nissaf Bouafif Ben Alaya, Afif Ben Salah

**Affiliations:** 1 Laboratory of Transmission, Control and Immunobiology of Infections (LR11IPT02), Institut Pasteur de Tunis, 13, Place Pasteur, BP 74, 1002 Tunis-Belvédère, Tunisia; 2 University of Carthage, Faculty of Sciences of Bizerte, Jarzouna, 7021 Bizerte, Tunisia; 3 National Institute of Public Health, 5-7 rue El-Khartoum, Tunis, Tunisia; 4 Faculty of Medicine of Tunis, University of Tunis El Manar, Tunis, Tunisia; 5 Observatoire National des Maladies Nouvelles et Emergentes de Tunis, Tunis, Tunisia; 6 Service of Medical Epidemiology, Institut Pasteur de Tunis, 13, Place Pasteur, BP 74, 1002 Tunis-Belvédère, Tunisia; 7 Primary Health Care Directorate, Tunis, Tunisia; 8 National Influenza Centre-Tunis, Unit Virology, Microbiology Laboratory, Charles Nicolle’s Hospital, Tunis, Tunisia; 9 Department of Infectious Diseases, Hedi Chaker University Hospital, Sfax, Tunisia; 10 Centers for Disease Control and Prevention, 1600 Clifton Rd NE, Mailstop A20, Atlanta, GA 30329-4027 USA; 11 Medical Intensive Care Unit Farhat Hached University Hospital, 4000 Sousse, Tunisia; 12 Research Laboratory, Heart Failure, N LR12SP09 Ibn Al Jazzar Faculty of Medicine, 4000 Sousse, Tunisia; 13 Non Invasive Ventilation Specialized Master Coordinator Ibn Al Jazzar, Faculty of Medicine, 4000 Sousse, Tunisia; 14 Pediatric Intensive Care Unit, Children’s Hospital Bechir Hamza of Tunis, Place Bab Saadoun, 1007 Tunis, Tunisia; 15 Intensive care department Abderrahmen Mami Hospital, Ariana, Tunisia; 16 Department of Family and Community Medicine, College of Medicine and Medical Sciences (CMMS), Arabian Gulf University (AGU), Manama, Bahrain

**Keywords:** Surveillance, Health system, Influenza, ILI, Evaluation, Tunisia

## Abstract

**Background:**

This study was initiated to evaluate, for the first time, the performance and quality of the influenza-like illness (ILI) surveillance system in Tunisia.

**Methods:**

The evaluation covered the period of 2012**–**2015 and used different data sources to measure indicators related to data quality and completeness, representativeness, timeliness, simplicity, acceptability, flexibility, stability and utility.

**Results:**

During the evaluation period, 485.221 ILI cases were reported among 6.386.621 outpatients at 268 ILI sentinel sites. To conserve resources, cases were only enrolled and tested for influenza during times when the number of patients meeting the ILI case definition exceeded 7% (10% after 2014) of the total number of outpatients for the week. When this benchmark was met, five to 10 patients were enrolled and sampled by nasopharyngeal swabs the following week. In total, The National Influenza Center (NIC) received 2476 samples, of which 683 (27.6%) were positive for influenza. The greatest strength of the system was its representativeness and flexibility. The timeliness of the data and the acceptability of the surveillance system performed moderately well; however, the utility of the data and the stability and simplicity of the surveillance system need improvement. Overall, the performance of the Tunisian influenza surveillance system was evaluated as performing moderately well for situational awareness in the country and for collecting representative influenza virologic samples.

**Conclusions:**

The influenza surveillance system in Tunisia provided pertinent evidence for public health interventions related to influenza situational awareness. To better monitor influenza, we propose that ILI surveillance should be limited to sites that are currently performing well and the quality of data collected should be closely monitored and improved.

## Background

Respiratory diseases remain a major cause of morbidity and mortality worldwide, with influenza infections being an important cause [1]. To better understand the epidemiology, including disease incidence and severity, a surveillance system must be implemented to collect data and inform public health authorities [2, 3]. The World Health Organization (WHO) established a global influenza surveillance network in 1952, which includes 136 National Influenza Centers in 106 countries [4]. Tunisia has been participating in the WHO global network through the Tunisian National Influenza Center, National Reference Influenza Laboratory (NIC), nominated and recognized by WHO since 1980. WHO recommends that influenza surveillance systems should be evaluated periodically, starting 1 to 2 years after their implementation in order to identify steps needed to fill gaps in performance [5]. This study is the first evaluation of the Tunisian influenza surveillance system since its implementation in 1980. We specifically aimed to: (i) provide a comprehensive understanding of how the Tunisian surveillance system operates; (ii) assess its performance and (iii) identify its strengths and weaknesses in order to improve it.

## Methods

### Description of the surveillance system

**Fig. 1:**
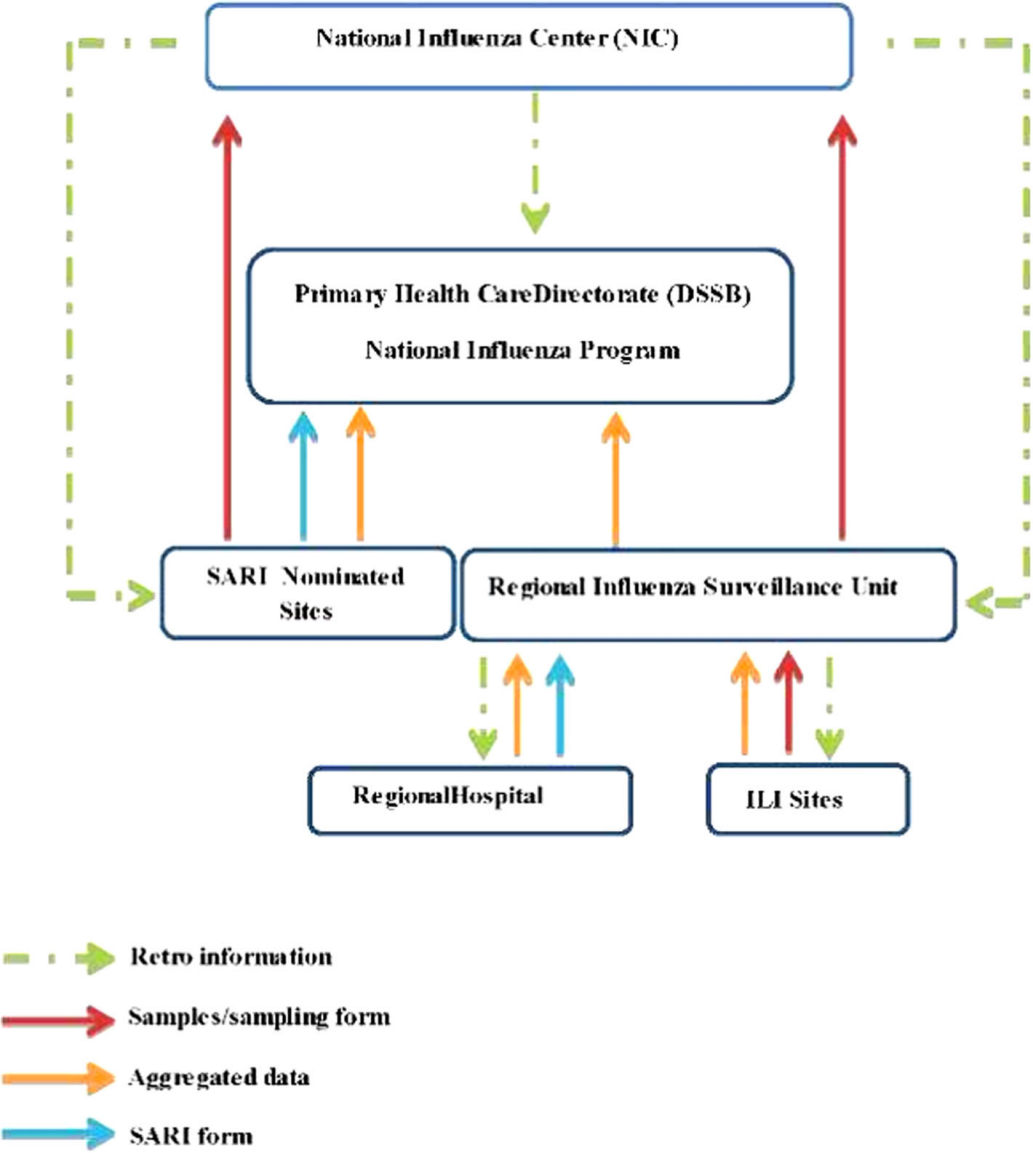
Structure of the influenza-like illness (ILI) surveillance system in Tunisia

The influenza surveillance system in Tunisia involves the following stakeholders: (i) Primary Health Care Directorate (DSSB), which coordinates the National Influenza Program at the Ministry of Health, (ii) the National Influenza Center (NIC) at the unit of virology, of the Microbiology Laboratory, Charles Nicolle’s Hospital, Tunis and (iii) a network of 268 sentinel surveillance sites selected from Primary Health Centers conducting influenza-like illness (ILI) surveillance (Fig. 1). The ILI sentinel sites were selected on the basis of their representativeness, their geographical–demographical criteria and the presence of voluntary and motivated health professionals. Since the start of surveillance, data was collected using standardized forms that aggregated data into three age groups: 0–5 years; 6–15 years and ≥ 16 years [6]. In 2014, however, a new WHO ILI case definition was implemented with minimum data standards that included gender and age groups (0- < 2 years, 2- < 5 years, 5- < 15 years, 15- < 50 years, 50- < 65 years and ≥ 65 years) [7]. ILI sites report cases from the 40th week of the year in October, to the 18th week of the following year in April, which correspond to the influenza season that started and finish in relation to the cold period of the northern hemisphere and the passage of migratory birds [8] (http://www.santetunisie.rns.tn/images/docs/anis/guidegripf6102016.pdf). The characteristics of the Tunisian influenza-like illness surveillance system are described in the Table 1.

**Table 1:** Characteristics of influenza-like illness surveillance system in Tunisia

Surveillance characteristics	ILI Surveillance
Syndrome	Influenza-like Illness (ILI)
Case definition before 2014	Outpatient with:
	• fever (38 °C)
	• and cough or sore throat
	• and onset less than five days prior to presentation
	• in the absence of a specific diagnosis
Case definition after 2014	Acute respiratory infection with:
	• measured fever (≥ 38 °C)
	• and cough
	• and onset within the past 10 days
Catchment population	Out-patient
Sites specialties	Primary Health Care Centers
Number of sentinel sites	Before 2014: 268 sites
	After 2014: 113 sites*
Geographic scope	24 governorates (all the country)
Coordinating body	Primary Health Care Direction:
	National Influenza Program
Specimen Collection	Nasopharyngeal swabs
Surveillance objectives	•Detection of influenza activity
	• Identification of circulating respiratory viruses
	• Identifying viruses for vaccine selection
	• Planning vaccination activities for prevention

*Based on the assessment of a national steering committee of expert that considered the staff motivation and the site effective contribution to surveillance without compromising representativeness

Samples are placed in a refrigerator at + 4 °C immediately after collection and transported in a virological transport medium to the NIC within 72 h. The NIC receives all of the nasopharyngeal swabs of sampled ILI cases to be tested for influenza and other respiratory viruses. At each sentinel site, if the number of patients meeting the ILI case definition exceeds 7% (10% after 2014) of the total number of outpatients (the total number of patients presented to the health care facility independently of the cause, this include all acute morbidity and excludes chronic diseases) for the week, randomly five to 10 patients responding the case definition were enrolled and sampled by nasopharyngeal swabs the following week [9]: this is the sampling criteria used [8].

(http://www.santetunisie.rns.tn/images/docs/anis/guidegripf6102016.pdf). Samples are weekly (5 working days from Monday to Friday) collected in order to detect influenza viruses, using real-time RT-PCR following the United States Centers for Disease Control and Prevention (US-CDC) recommended diagnostic using validated in house protocols [10]. The NIC communicates the results to the sentinel sites, the DSSB and the WHO FluNet global monitoring system. The DSSB is responsible for the coordination of disease surveillance and response and ensures the reporting at national and international levels.

### Evaluation of the influenza surveillance system

The evaluation period included three influenza seasons from October 2012 to April 2015.The strengthening project of the influenza surveillance system starts on 2014. So we decided to evaluate the year of the new strategy implementation with two years before the project (pre-evaluation) to have a clear idea about the system performance at time zero, just when we started the new version of the surveillance system and to evaluate the changes in the future after the end of the project (post-evaluation). Surveillance system performance was assessed using the guidelines of the US-CDC [11]. Eight attributes were considered: (i) data quality and completeness; (ii) representativeness; (iii) timeliness; (iv) simplicity; (v) acceptability; (vi) flexibility; (vii) stability and (viii) utility. For each attribute, specific indicators were developed. Indicators were analyzed, scored and a mean score was calculated for each attribute using a qualitative scale from 1 to 5, from very poor performance (1) to very good performance (5). In this scale, we have collapsed two categories for some questions: score 1 (strongly agree / agree); score 2 (mostly agree); score 3 (somewhat disagree); score 4 (Disagree/ strongly disagree) and score 5 (Do not know). We used surveillance databases collected at the National Surveillance Program of the Ministry of Health and the NIC. Standardized semi-structured and self-administered questionnaires were used to collect relevant data from surveillance staff at ILI sites, regional directorates, the DSSB and from the NIC.

## Results

During the evaluation period, 485.221 ILI cases were reported among 6.386.621 outpatients at 268 ILI sentinel sites. In total, 2476 samples were tested, of which 683 were positive (positivity rate 27.6%). During these three seasons, the distribution of influenza viruses (influenza B, influenza A (H1N1) pdm2009 and influenza A (H3N2)) varied, with different subtypes predominant in each season (Table 2).

**Table 2:** Descriptive results of the influenza-like illness surveillance during three influenza seasons in Tunisia, 2012–2015

	2012–2013	2013–2014	2014–2015	Total
Number of outpatients	2.023.942	2.196.715	2.165.964	6.386.621
Number of ILI cases	170.623	156.513	158.085	485.221
Samples tested	924	514	1038	2476
Proportion of positive samples for Influenza (%)	37.4	12.1	28.0	27.6
Virus A(H3N2) (%)	6.1	96.8	15.5	18.4
Virus A(H1N1) pdm2009 (%)	50.1	1.6	39.2	42.9
Virus B (%)	38.3	1.6	45.3	38.7
Unsubtyped viruses (%)	5.5	/	/	/

The self-administered questionnaires were administered to 599 surveillance staff at ILI sites (from which 3.2% (19/599) were discarded due to missing data), 85 at regional directorates, six at the DSSB and seven at the NIC. Table 3 presents the mean scores of the eight assessed attributes as well as the scores of all the indicators that were used for the evaluation of the influenza surveillance system in Tunisia.

**Table 3:** Findings from the evaluation of the influenza like-illness surveillance system in Tunisia, 2012–2015

Attributes and definitions	Indicators	Scores ^a^	Mean score
Data quality and completeness The completeness and validity of the data recorded in the public health surveillance system	• Proportion of ILI surveillance staff that identified correctly the ILI case definition	2	2.7
	• Proportion of ILI surveillance staff that identified correctly the sampling criteria	2	
	• Proportion of collected variables included in the WHO recommended minimum data collection for influenza sentinel surveillance^b^	2	
	• Quality and proficiency of NIC laboratory detection of of influenza using RT-PCR	5	
Representativeness Describes the occurrence of a health-related event over time and its distribution in the population by place and person	• Geographical coverage ^d^	4	4.5
	• Inclusion of all age groups ^d^	5	
Timeliness Reflects the speed between steps in a public health surveillance system	• Proportion of NIC staff estimating that more than 80% of results of tested samples were obtained within 7days from the date of reception	5	3.4
	• Proportion of ILI surveillance staff estimating that more than 80% of aggregated data were sent within 7 days from ILI sites to regional directorates	3	
	• Proportion of ILI surveillance staff estimating that more than 80% of aggregated data were sent within 1 month from ILI sites to regional directorates	3	
	• Proportion of regional directorates surveillance staff estimating that more than 80% of aggregated data were sent within 7 days to DSSB	3	
	• Proportion of regional directorates surveillance staff estimating that more than 80% of aggregated data were sent within 1 month to DSSB	3	
Simplicity Refers to both structure and ease of operation of a public health surveillance system	• Perception of surveillance staff on the ease of accomplishing these surveillance activities:	2	2.5
	▪ Data collection for the sampling form	3	
	▪ Data collection for the shipment form	3	
	▪ specimen collection	2	
	• Mean of time devoted to weekly surveillance activities ^c^		
Acceptability The willingness of persons and organizations to participate in the surveillance system	• Proportion of surveillance staff that was satisfied with the following:		3.0
	▪ Virological surveillance report	2	
	▪ Influenza bulletin	2	
	▪ Communication	3	
	• The proportion of surveillance staff that reported that the surveillance system was good:		
	▪ ILI surveillance staff	3	
	▪ NIC surveillance staff	4	
	▪ Regional directorate	4	
Flexibility The ability of a surveillance system to changing information needs or operating conditions with little additional time, personnel, or allocated funds	• The 2014 decrease in the number of ILI sites performing surveillance ^d^	3	4.0
	• The adoption of new ILI forms ^d^	4	
	• Inclusion of other pathogens surveyed with influenza surveillance system ^d^	5	
Stability The reliability and availability of the public health surveillance system	• Proportion of ILI surveillance staff that report using:		2.7
	▪ SOP for sampling	3	
	▪ SOP for shipment	3	
	▪ Influenza Surveillance Guide	2	
	• Proportion of ILI surveillance staff that report being trained on:		
	▪ Epidemiological surveillance	2	
	▪ Influenza surveillance activities	2	
	▪ Influenza-specific response activities	2	
	▪ The practice of nasopharyngeal specimens	2	
	• Proportion of ILI surveillance staff that reported that depletion of stock never occurred for:		
	▪ Data collection forms	4	
	▪ Sampling material	3	
	▪ Protective equipment	3	
Utility Does the system provide information that is useful for public health authorities and communities	• Proportion of ILI surveillance staff that reported that the influenza surveillance system:		3.6
	▪ was important	4	
	▪ provided useful data	4	
	• Proportion of ILI surveillance staff that reported that they regularly receive the following reports:		
	▪ Virological surveillance report	2	
	▪ Monthly Influenza bulletin	2	
	▪ Annual Influenza report	3	
	• Identification and sharing of circulating seasonal influenza strains^d^	5	
	• Contribution of influenza viruses to WHO CC for vaccine strain selection:		
	▪ participation with WHO CC for vaccine selection	5	
	▪ number of shipments	4	
	▪ adherence to recommended timing of shipment	3	
	• Outbreaks detected over pre-established threshold during the evaluation period ^d^	4	
Overall total			3.3

*ILI*: influenza like-illness, *WHO*: World Health Organization, *NIC*: National Influenza Center, *DSSB*: Primary Health Care Directorate, *SOP*: Standard operating procedures, *WHO CC*: WHO Collaborating Centers on influenza

a: a scale from 1 to 5 was used to provide a score for each indicator with a percentage value as follows: [0–20%[score 1 (very poor performance); [20–40%[score 2 (poor performance); [40–60%[score 3 (moderate performance); [60–80%[score 4 (good performance); [80–100%[score 5 (very good performance)

b: The Tunisian surveillance system lacks data, within the list of the recommended WHO minimum data, on body temperature at presentation, date of symptom onset, date of specimen collection, seasonal influenza vaccination status, antiviral treatment and underlying medical conditions

c: a scale from 1 to 5 was used to provide a score for the mean of time devoted to weekly surveillance activities with a percentage value as follows: [1–5%[score 5 (very good performance); [5–10%[score 4 (good performance); [10–15%[score 3 (moderate performance); [15–20%[score 2 (poor performance); [20–25%[score 1 (very poor performance)

d: These indicators were scored from 1 to 5 but these scores were based on the consensus opinions of surveillance experts: virologist, public health specialist and epidemiologists as follows: score 1 (very poor performance); score 2 (poor performance); score 3 (moderate performance); score 4 (good performance) and score 5 (very good performance)

### Data quality and completeness

Among ILI surveillance staff, 30% (*n* = 174/580) correctly identified the ILI case definition and 35.8% (*n* = 208/580) correctly indicated the sampling criteria for influenza. We were not able to determine the number of samples swabbed for influenza viruses at each ILI sites, because of lack of individual identification of each ILI site in the previous surveillance system (see also Table 3). In addition, the aggregated data from the variables on case report forms were not complete at the central level and aggregated data was not routinely sent to the regional directorates. If we compare with the WHO minimum data requirements [12] (https://ecdc.europa.eu/en/seasonal-influenza/surveillance-and-disease-data/facts), the proportion of recommended variables that are collected within the WHO list was 36.6%. Results from the WHO External Quality Assurance Program (EQAP) show participation of the NIC in all evaluation years with a perfect performance (100% in each year of evaluation). Therefore, from a virologic perspective, once samples are received by the national influenza lab, there is evidence of quality and proficiency of RT-PCR diagnosis of influenza and accuracy of lab testing method. The mean score for data quality was 2.7 (poor to moderate performance).

### Representativeness

ILI surveillance is conducted in both rural and urban outpatient clinics (approximately equally distributed), situated in all the 24 governorates of Tunisia. The number of sentinel sites per governorate is proportional to the population size. The distribution of age groups was representative of the population. The mean score for representativeness was 4.5 (good to very good performance).

### Timeliness

Because of the high proportion of missing data regarding date of specimen collection, reception and analysis at the NIC, an indicator of data quality, we were unable to directly assess timeliness. We used qualitative questionnaires to assess sentinel site staff perceptions of timeliness. All NIC staff (*n* = 7/7) perceived that over 80% of specimens tested had results available within seven days of receipt at the laboratory. Almost half of ILI and regional directorate health workers interviewed reported that more than 80% of aggregated data forms were sent within seven days (55.8% [*n* = 324/580] and 44.1% [*n* = 37/85], respectively). In addition, 46.1% (*n* = 267/580) of ILI surveillance staff and 52.0% (*n* = 44/85) of regional directorates’ staff reported that more than 80% of aggregated data forms were sent within one month. The mean score for timeliness was 3.4 (moderate to good performance).

### Simplicity

In this study, simplicity was assessed using a questionnaire to evaluate staff perception of how easy it was to perform tasks related to data and sample collection, and how much time it took per week. The proportion of healthcare workers at ILI sites that reported the data collection for the sampling form, shipment forms and sampling practice was considered as easy was 35.1% (*n* = 204/580), 53.4% (*n* = 310/580) and 53.5% (n = 310/580), respectively. The mean time devoted to weekly activities on ILI sentinel surveillance sites was 17.5% of total working hours per week (7 h/40-h week). The mean score for simplicity was 2.5 (poor to moderate performance).

### Acceptability

Acceptability was measured by the satisfaction of surveillance staff with the reports generated using the information they provided. The proportion of surveillance staff who were satisfied by the virological surveillance report, the influenza bulletin and communication at ILI sites was 45.5% (*n* = 264/580), 57.6% (*n* = 334/580) and 65.9% (*n* = 382/580), respectively. The system was rated as globally good by 40% (*n* = 170/424), 71.4% (*n* = 5/7) and 60.4% (*n* = 63/85) of health professionals at ILI sites, NIC and regional directorates, respectively. The mean score for acceptability was 3.0 (moderate performance).

### Flexibility

After 2014, the reduction of the number of ILI sites from 268 to 113 realized to improve the chances of higher data quality without compromising the representativeness demonstrate the flexibility of the system. Furthermore, after 2014, new ILI case report forms were prepared and distributed to all stakeholders of the surveillance system taking into account WHO recommendations. Over the years, the capacity and flexibility of the NIC also increased, from the ability to detect influenza A(H3N2), A(H1N1) and B to influenza A(H1N1)pdm09 and avian influenza virus subtypes A(H5N1), A(H7N9) and A(H9N2). The NIC’s ability to detect non-influenza pathogens also reflects the system flexibility. Other respiratory viruses are detected within the routine ILI surveillance system including RSV, adenovirus, enterovirus/rhinovirus, metapneumovirus and parainfluenza virus [9]. The discovery of a familial cluster of Middle East Respiratory Syndrome Coronavirus in 2013 confirmed the importance and flexibility of this surveillance system [13]. The mean score for flexibility was 4.0 (good performance).

### Stability

Approximately half of the surveillance staff at ILI sites reported the regular use of standard operating procedures for sampling (50.7%, *n* = 294/580), shipment forms (53.4%, *n* = 310/580) and Influenza Surveillance Guide (54.0%, *n* = 313/580). We assessed training offered to surveillance staff. Less than half of surveillance staff were trained on epidemiological surveillance (33.4%, *n* = 194/580), influenza surveillance activities (28.0%, *n* = 162/580), influenza-specific response activities (24.0%, *n* = 139/580) and the practice of nasopharyngeal specimen collection (33.4%, n = 194/580). Resource depletion events were also assessed. Surveillance staff reported that depletion of stock never occurred for: data collection forms (65%, *n* = 377/580), specimen collection materials (56.2%, *n* = 326/580) and personal protective equipment (53.7%, *n* = 311/580). The mean score for stability was 2.7 (poor performance to moderate performance).

### Utility

The majority of surveillance staff recognized the importance of an influenza surveillance system (70.4%, *n* = 408/580) and thought that it provided useful data (78.5%, *n* = 455/580). Only 37.9% (*n* = 220/580) of surveillance staff reported regular receipt of the virological surveillance reports and the monthly influenza bulletin (42.9%, *n* = 249/580). Over half of staff (57.1%, *n* = 331/580) reported never receiving the annual influenza report.

**Fig. 2:**
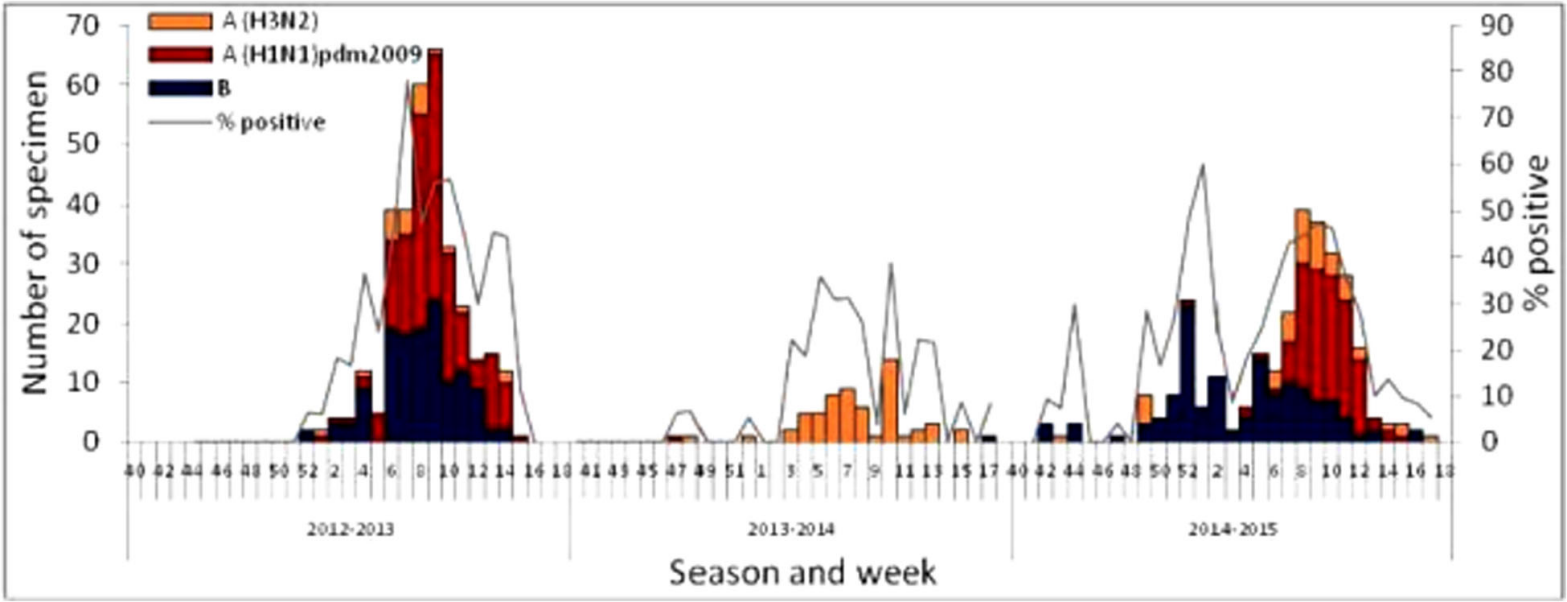
Seasonal influenza virus detection by type and subtype in Tunisia, 2012–2015

From a virologic perspective, the surveillance of influenza between 2012 and 2015 permitted the characterization of circulating influenza viruses (Fig. 2). Furthermore, the NIC ensured strong links with the WHO Collaborating Centers on influenza by participating in vaccine strain selection and shipping specimens twice a year as recommended by WHO. The system was also useful to detect an outbreak with medium intensity during the 2012–2013 season and an increase of influenza activity above the epidemic threshold during week 8 in 2014–2015 [14]. Additionally, national surveillance data allowed the calculation of the burden of disease for the first time in 2016 [6]. The mean score for utility was 3.

The overall mean score of the influenza surveillance system in Tunisia, considering the eight components described above was 3.3, indicating a moderate performance.

## Discussion

This study assessed the Tunisian influenza surveillance system for the first time, to provide a comprehensive summary of its process, strengths and weaknesses. The performance of the current system was moderate overall (mean score 3.3) and representativeness was a strength, with a mean score of 4.5 (good to very good performance).

The system has also demonstrated a strong flexibility, with a mean score of 4.0 (good performance). To make use of the flexible system, we recommend to continue monitoring other diseases using the same surveillance system to increase efficiency and avoid creation of additional vertical surveillance programs.

We were not able to score indicators that directly measure the time between specimen collection and laboratory results, which is a source of bias in evaluating the surveillance system. Measuring timeliness was not possible due to the incompleteness in reporting dates of collection, receipt, testing, and reporting. In this example, gaps in completeness of data affected the ability to objectively assess quality and timeliness of data. The timeliness of the system may be affected by the geographical distribution of sentinel sites and the simplicity of data collection and reporting methods. An electronic information management system was one of the most important recommendations proposed by surveillance staff to improve data completeness, quality monitoring and timeliness. It would facilitate real-time sharing of data [15]. A recent evaluation of a syndromic influenza sentinel surveillance system in Madagascar between 2009 and 2014 showed that by using mobile phones and texting for the transmission of daily aggregated data, the simplicity of the system was strengthened, improving the completeness, quality and timeliness of the data and the acceptability of the system to the sentinel site staff [16]. This is in line with the recommendation that a revised data management approach, which offers the advantage of automation and user-friendly interface, would ultimately improve the acceptance and utility of the surveillance system [17]. Utility and acceptability of the system were also moderate, with a mean score of 3.6 and 3.0, respectively.

It is clear that even with the strengths identified, the system also presents some weaknesses, mainly regarding its stability and simplicity, with mean scores of 2.7, 2.5, respectively (moderate to poor performance). The stability of the system is affected by access to materials, training and resources required by staff who conducts surveillance and influenza testing. This is an area of vulnerability, as current material resources are limited, and may decrease acceptability of the system among staff. Moreover, one of the mechanisms to strengthen the surveillance system is to train at least two persons per site and to ensure cascade training in the sites in every season to avoid the consequences of qualified staff departure.

The simplicity also affects the data quality and completeness, which was also identified as a weakness, with a mean score of 2.7 (moderate to poor performance). The aggregated data were incomplete for key variables such as the number of samples tested for influenza at individual ILI sites and several sites were not consistently sending this information to the regional directorates. We recommended enhancement of awareness and sensitivity of surveillance staff regarding the importance of key variables and to strengthen training on data collection and reporting. Inclusion of ILI sites in the influenza surveillance system should be revisited periodically by assessing timely reporting and quality and completeness of data collected. Moreover, an electronic information management system would improve completeness of key variables. On the other hand, the surveillance system presents a strong point in virologic surveillance internationally confirmed by the results from the WHO EQAP and participation in global influenza vaccine strain selection and WHO FluNet virologic monitoring. The foremost importance of the surveillance system is to detect the viruses causing and their activities for influenza-like illness rather than influenza. Improving the quality and completeness of virologic sampling and reporting is essential to guarantee the identification of circulating viruses and their local seasonality in Tunisia. The early detection and characterization of these viruses allow timely annual updates for a vaccine that can prevent deaths and alleviate illness in vulnerable groups. Sensitivity, specificity and positive predictive value were not included in this evaluation because of a lack of data. It is recommended to assess these attributes in the next surveillance system evaluation.

## Conclusions

Many gaps still exist and require attention to effectively guide future control of influenza in Tunisia. In line with WHO recommendations [7], we propose that ILI surveillance should be limited to sites that are currently performing well as part of the network and the improvement of data and sample collection to be more systematically gathered. This evaluation provides a good opportunity to monitor future improvements in influenza surveillance, by quantifying evaluation indicators prior to and after the implementation of a new strategy based on quality improvement and electronic surveillance. Future evaluations are highly recommended to measure the impact of such strategies on the performance and sustainability of the surveillance system.

## Abbreviations

DSSB: Primary Health Care Directorate
EQAP: External Quality Assurance Program
ILI: Influenza-like illness
NIC: National Influenza Center
US-CDC: United States Centers for Disease Control and Prevention
WHO: World Health Organization
